# On the Single-Point Calculation of Stress–Strain Data under Large Deformations with Stress and Mixed Control

**DOI:** 10.3390/ma15196644

**Published:** 2022-09-25

**Authors:** Mingchuan Wang, Cai Chen

**Affiliations:** Sino-French Engineer School, Nanjing University of Science and Technology, Nanjing 210094, China

**Keywords:** constitutive model, large deformation, stress–strain data, stress control, crystal plasticity, particle reinforce composite

## Abstract

Stress–strain data with a given constitutive model of material can be calculated directly at a single material point. In this work, we propose a framework to perform single-point calculations under large deformations with stress and mixed control, to test and validate sophisticated constitutive models for materials. Inspired by Galerkin–FFT methods, a well-defined mask projector is used for stress and mixed control, and the derived nonlinear equations are solved in Newton iterations with Krylov solvers, simplifying implementation. One application example of the single-point calculator in developing sophisticated models for anisotropic single crystal rate-independent elastoplasticity is given, illustrating that the proposed algorithm can simulate asymmetrical deformation responses under uni-axial loading. Another example for artificial neural network models of the particle reinforced composite is also given, demonstrating that the commonly used machine learning or deep learning modeling frameworks can be directly incorporated into the proposed calculator. The central difference approximation of the tangent is validated so that derivative-free calculations for black-box constitutive models are possible. The proposed Python-coded single-point calculator is shown to be capable of quickly building, testing, and validating constitutive models with sophisticated or implicit structures, thus boosting the development of novel constitutive models for advanced solid materials.

## 1. Introduction

Constitutive models are important mathematical tools to describe material responses to complex mechanical loading conditions. With increasing demands for studying in depth and simulating deformations of different types of advanced materials [1,2,3,4], their corresponding constitutive models become increasingly complex. For example, crystal plasticity models for metal materials [5] under large deformations describe several microscopic deformation mechanisms, such as dislocation motions, twinning, and phase transformation, using multiple internal variables, corresponding equations, and even more than two loops of iteration procedures to build a sophisticated model structures [6]. Constructing an advanced constitutive model is challenging even for well-trained researchers. In addition, a developed complex constitutive model needs to be tested with experimental results or microscopic calculations, before it is regarded as a reliable model. Thus, it is not trivial to have a simple calculation framework to test advanced constitutive models, debug the program for models, and facilitate parameter calibration [7]. In addition, a potential application of the sophisticated constitutive models is to generate microscopic calculation data, for macroscopic model-free data-driven mechanics [8] or for establishing macroscopic data-driven machine learning models [9,10,11,12,13]. Thus, a calculation framework that quickly calculates and outputs data for demanded field variables (e.g., stress–strain data) is highly recommended.

To implement one constitutive model, a popular method is to code the model as one subroutine to the program of finite element methods (FEM), such as ABAQUS [14,15,16,17,18] and LS-DYNA [19,20,21]. To validate the constitutive model, the structure simulation or one-element calculation is performed in FEM programs with the constitutive model subroutines and then compared with experimental results or microscopic calculations. For FEM simulations, nonlinear geometries, nonlinear boundary conditions, and chosen interpolation functions in elements may produce extra calculation errors, which sometimes need much more effort to overcome than a nonlinear constitutive model does. To validate constitutive models or calibrate parameters, different paths of deformation loading for one material point are needed to benchmark the models. In that case, a single-point calculator can be applied as an alternative but more efficient calculation framework to FEM.

The single-point calculator runs in a single material point, calculating the stress response with the prescribed strain directly with the constitutive model. For example, when a simple shear is simulated, a prescribed deformation gradient can be given in a form (γ≠0) as follows:

$$\bar{F}=\left[\begin{array}{ccc} 1 & \gamma & 0\\ 0 & 1 & 0\\ 0 & 0 & 1 \end{array}\right],$$

indicating no change of volume, as its determinant equals one. The  stress can then be calculated directly in the single-point calculator, with a chosen strain measurement calculated from this prescribed deformation gradient. Calculating stress directly from the strains is called the problem of strain control. However, the inverse problem in the single-point calculator, calculating the strain from the stress, is not direct. It is often impossible to inverse advanced constitutive models, which use complicated mechanisms to calculate the stress from the strain. In addition, in some cases of loading, for example, uni-axial loading, mixed components of stress and strain are given in the single-point calculator to determine the other components. Especially for the anisotropic incompressible solid whose determinant of deformation gradient equals 1, the components of the deformation gradient corresponding to the other two dilatation directions are hard to prescribe accurately and directly in the case of uni-axial loading [22]. The problem to solve is to calculate the deformation gradient or the missing components of the stress/deformation gradient with given stress or mixed components, which corresponds to the problem of stress and mixed control. Therefore, to extend the applications of the single-point calculator, the stress and mixed control boundary conditions must be considered.

Inspired by the Galerkin–FFT methods [23,24,25], we propose an algorithm for the problem of stress and mixed control in the single-point calculator. Analogously to the algorithm for stress and mixed control in Galerkin–FFT methods [25,26], our calculator uses a well-defined mask projector to maintain a unified form of core equations to solve for strain control, stress control, or mixed control. As in the Galerkin–FFT methods, the core equations are to be solved with a Krylov solver, such as conjugate gradient (CG) [24,27] and minimum residual (MinRes) [28], which allows circumventing the difficulties associated with the singularity of the tangent stiffness tensor. These two characteristics (the usage of a mask projector and a Krylov solver) of our proposed algorithm make the calculator simple to implement. Details of the algorithm are given in Section 2. The proposed single-point calculator and the associated constitutive models are recommended to be coded in Python, to facilitate the procedure of testing new algorithms, validating sophisticated mathematical structures, and identifying parameters for constitutive models. With the built-in scientific calculation package, Numpy and Scipy [29], the speed of calculation with Python is acceptable even for industrial demands. Two examples of applications of the single-point calculator for the development of constitutive models are given in Section 3. The first example for anisotropic single crystal plasticity (Section 3.1) illustrates that the proposed calculation framework is simple to develop models with sophisticated algorithms and mathematical structures. The second example of the artificial neural network elasticity model (Section 3.2) illustrates the single-point calculator’s compatibility with machine learning methods or deep learning methods [30], which can be implemented in commonly used Python-entranced frameworks, such as TensorFlow [31] or PyTorch [32]. Even though the exact analytical consistent tangent is hard to obtain for implicit networks of constitutive models, the central difference approximation of the tangent [33,34] can be used in the single-point calculator. The derivative-free calculations for black-box constitutive models are validated in the second example.

*Notation.* In this work, scalars, second-order tensors, and fourth-order tensors are denoted as *s*, T, and K, respectively. The double dot product between two second-order tensors is denoted as P:F and calculated as $R=P_{ij}F_{ij}$ (i,j=1,2,3), where the Einstein summation convention is used. The double dot product between one fourth-order tensor and one second-order tensor is denoted as C:e and calculated as $R_{ij}=C_{ijmn}e_{mn}$. The operator ${\left(*\right)}^{T}$ denotes the transpose, while ${\left(*\right)}^{-1}$ means the inverse of a tensor. The operator det(*) represents the determinant of the matrix corresponding to the tensor.

## 2. The Single-Point Calculator

### 2.1. Strain Control in the Single-Point Calculator

We consider two conjugated stress and strain tensors, the first Piola–Kirchhoff stress P and the deformation gradient F. For the strain control problems, the single-point calculator is a numerical tool to calculate the corresponding values of P from F with a given constitutive model. If values of F corresponding to finite points in the loading path are given, the single-point calculator can simply calculate the stress responses P one by one. When the prescribed tensor is divided into *N* segments, the *n*-th stress response is calculated with the *n*-th deformation gradient as follows:

(1) $$P^{\left(n\right)}=P\left(F^{\left(n\right)}\right)\;\;,\;\;n\in \left(1,2,...,N\right).$$

Equation (1) can be used for the elasticity-like solids. As for the materials depending on the loading history, such as visco-elastic or plastic solids, the rate of the deformation gradient is also needed. We can use the deformation gradient in the former step to calculate the current step:

(2) $$P^{\left(n\right)}=P\left(F^{\left(n\right)},F^{\left(n-1\right)},\Delta t,\alpha^{\left(n-1\right)},...\right),$$

where Δt is the time increment and α represents internal variables. Here, the rate of the deformation gradient can be approximated as follows:

(3) $${\dot{F}}^{\left(n\right)}=\frac{F^{\left(n\right)}-F^{\left(n-1\right)}}{\Delta t}.$$

However, the rate of deformation gradient is often replaced by the velocity gradient, which can be approximated as follows:

(4) $$L^{\left(n\right)}=\frac{F^{\left(n\right)}-F^{\left(n-1\right)}}{\Delta t}{\left(\frac{F^{\left(n\right)}+F^{\left(n-1\right)}}{2}\right)}^{-1}.$$

Compared with reliable experimental results of the same loading path, the calculated responses can be used to validate the developed constitutive model (Equations (1) and (2)) or to identify model parameters.

### 2.2. Stress and Mixed Control in the Single-Point Calculator

When the single-point calculator is controlled by the strain, i.e., calculating the stress with the prescribed deformation gradient, the calculation is direct and simple. However, an iterative algorithm is needed to solve the inverse responses, in the case of stress and mixed control. For example, the mixed prescribed components of stress and the deformation gradient are given:

$${\bar{F}}_{ij},\;\;{\bar{P}}_{IJ},\;\;ij\in A,\;\;IJ\in B,\;\;A\cap B=⌀,\;\;A\cup B=\left(11,12,13,21,22,23,31,32,33\right),$$

where A denotes the set of indices of components of the prescribed deformation gradient, while B denotes those of the prescribed stress. The  corresponding components of the stress and the deformation gradient, $F_{IJ}$ and $P_{ij}$, are unknowns to be solved. It is noted that nine components in different positions are needed in order to obtain unique responses to the problem. In fact, only the unknown components of the deformation gradient $F_{IJ}$ are to be solved in the iteration algorithm. Once all of the components of the deformation gradient are known, the components of stress can be easily calculated with Equation (1) or Equation (2).

Analogously to the algorithm for stress and mixed control in the Galerkin–FFT methods [25,26], we define a mask operator N:

(5) $$N_{ijmn}=\left\{\begin{array}{cc} 0_{ijmn} & \mathrm{if}\;\;mn\in A\\ \delta_{im}\delta_{jn} & \mathrm{if}\;\;mn\in B \end{array}\right.,$$

where δ is the Kronecker delta. Thus, it can project an arbitrary tensor into its masked counterpart, such that only the components in the position corresponding to the stress control are not zeros:

(6) $$N_{ijmn}A_{mn}=\left\{\begin{array}{cc} 0_{ij} & \mathrm{if}\;\;ij\in A\\ A_{ij} & \mathrm{if}\;\;ij\in B \end{array}\right..$$

Suppose that the components of the *n*-th deformation gradient are prescribed as follows:

(7) $${\bar{F}}_{ij}^{\left(n\right)}=\left\{\begin{array}{cc} {\bar{F}}_{ij}^{\left(n\right)} & \mathrm{if}\;\;ij\in A\\ 0_{ij} & \mathrm{if}\;\;ij\in B \end{array}\right..$$

The *n*-th deformation gradient in the loading path can be separated into two parts:

(8) $$F^{\left(n\right)}={\bar{F}}^{\left(n\right)}+N:\tilde{F}$$

where $\tilde{F}$ is an arbitrary deformation gradient increment. If the *n*-th deformation gradient $F^{\left(n\right)}$ and the stress $P^{\left(n\right)}$ are known, the unknown components of the deformation gradient and the stress in Step n+1, where an iterative algorithm is applied, can be determined. The iteration equation is established by linearizing the expression of the stress with Equations (1) and (8):

(9) $$P^{\left(k+1\right)}=P\left(F^{\left(k+1\right)}\right)=P\left(F^{\left(k\right)}+N:\delta {\tilde{F}}^{\left(k\right)}\right)\approx P^{\left(k\right)}+K^{\left(k\right)}:N:\delta {\tilde{F}}^{\left(k\right)},$$

where the fourth-order tensor K denotes the consistent tangent, K=∂P/∂F. The admissible condition for the stress is that the components should be equal to those prescribed values:

(10) $$N:P^{\left(k+1\right)}=N:{\bar{P}}^{\left(n+1\right)}.$$

Substituting Equation (9) into Equation (10), we arrive at the system of linear equations to be solved in the procedure of iterations:

(11) $$N:K^{\left(k\right)}:N:\delta {\tilde{F}}^{\left(k\right)}=N:\left({\bar{P}}^{\left(n+1\right)}-P^{\left(k\right)}\right),$$

where the increment of an arbitrary deformation gradient $\delta {\tilde{F}}^{\left(k\right)}$ is the only unknown.

### 2.3. Implementation of the Single-Point Calculator

The procedure of the single-point calculator is described as follows:

(1) Divide the loading into *N* segments. Thus, the prescribed deformation gradient and stress increase in *N* steps accordingly.
(2) Calculate the deformation gradient and the stress with the known tensors in the last step and the prescribed values in this step. The initial deformation gradient is set as $F^{\left(0\right)}=I$, and the initial stress can be set as $P^{\left(0\right)}=0$.
(3) Perform the Newton iteration within one step, as shown in Algorithm 1. In the (k+1)-th iteration, the increment of the deformation gradient can be solved from Equation (11). The deformation gradient is then updated:
  (12) $$F^{\left(k+1\right)}=F^{\left(k\right)}+N:\delta {\tilde{F}}^{\left(k\right)}.$$
  The stress is updated: $P^{\left(k+1\right)}=P\left(F^{\left(k+1\right)}\right)$. The iteration goes on until the acceptable deformation gradient and stress are found.

The algorithm of the single-point calculator is implemented in Python. The coded Python program only uses scientific calculation packages Numpy and Scipy. The codes of the single-point calculator are available online (https://gitee.com/withmc/spc, accessed on 19 septembre 2022). The system of linear Equations (Equation (11)) of the single-point calculator is solved with Krylov solvers, such as CG or MinRes from Scipy, which are also the core solvers for the Galerkin–FFT methods. Even if the consistent tangent in Equation (11) is singular, the MinRes solver is able to find relevant solutions [28]. Before and during the Newton iteration of finding increments of the deformation gradient, Step 4 in Algorithm 1, the studied constitutive model is called to calculate the stress P and the tangent stiffness K. It is noted that the iteration is not necessary when the problem is only strain control, as the components of the mask operator N are zeros.
**Algorithm 1** Iteration in the stress and mixed control single-point calculator.**procedure**Given $F^{\left(n\right)}$ and ${\bar{F}}^{\left(n\right)}$, ${\bar{F}}^{\left(n+1\right)}$, ${\bar{P}}^{\left(n\right)}$, ${\bar{P}}^{\left(n+1\right)}$, Calculate $F^{\left(n+1\right)}$ and $P^{\left(n+1\right)}$    (1) $F^{\left(0\right)}=F^{\left(n\right)}+{\bar{F}}^{\left(n+1\right)}-{\bar{F}}^{\left(n\right)}$    **while** $|N:\delta \tilde{F}|>\epsilon_{1}$ or $|N:\left(P-\bar{P}\right)|>\epsilon_{2}$ **do**        (2) Calculate $N:\delta \tilde{F}$ with Equation (11).        (3) Update F with Equation (12).        (4) Calculate P and K with the constitutive model.    **end while**    (5) Accept the converged results: $F^{\left(n+1\right)}=F$, $P^{\left(n+1\right)}=P$**end procedure**Note: $\epsilon_{1}$ and $\epsilon_{2}$ are two positive values chosen as approximations to zero.

## 3. Applications of the Single-Point Calculator

In this section, two examples of applications of the single-point calculator are introduced. The first example illustrates the calculator’s capability of dealing with a complicated constitutive model for incompressible and anisotropic single crystal plastic solids. The second example illustrates that the Python coded calculator is able to build machine learning constitutive models directly and simply.

### 3.1. Application in Tests of a Single Crystal Plasticity Model

As the first example, a recently proposed advanced model for single crystal plasticity [18] is tested with the single-point calculator.

#### 3.1.1. Constitutive Model

A short review of the model is presented first. A single crystal plasticity model is path-dependent, so its constitutive model is in the form of Equation (2). When the constitutive model is called, the deformation gradient F in the current iteration, the deformation gradient $F_{0}$ and the internal variables $\alpha_{0}$ in the last increment are given, in order to calculate the current stress P, the consistent tangent K, and the updated internal variables. For this single crystal plasticity model, the internal variables are the Cauchy stress σ, the total accumulated slip Γ, the rate of slip in each slip system ${\dot{\gamma }}^{\left(\alpha \right)}$, the critical resolved shear stress $\tau_{c}^{\left(\alpha \right)}$, and the orientation matrix Q. The index α takes values from 1 to the number of slip systems $N_{s}$.

Given the current and the last deformation gradients, we can calculate the velocity gradient L with Equation (4). The symmetric and the skew-symmetric part of the velocity gradient are used in constructing the model. They are called the strain rate and the spin, respectively:

(13) $$D=\frac{1}{2}\left(L+L^{T}\right)\;\;,\;\;W=\frac{1}{2}\left(L-L^{T}\right).$$

An increment of rotation can be calculated with the spin [35] as follows:

(14) $$\Delta R={\left(I-\frac{1}{2}W\Delta t\right)}^{-1}\left(I+\frac{1}{2}W\Delta t\right).$$

A trial orientation matrix $Q_{\mathrm{trial}}$ can then be updated from the last orientation matrix $Q_{0}$ with the increment of rotation:

(15) $$Q_{\mathrm{trial}}=\Delta R^{T}Q_{0}$$

The initial orientation matrix is determined by the orientation of the crystal before the deformation. In this single crystal plasticity model, the stress and the related internal variables are updated in the co-rotational frame or the lattice frame, so that the lattice tensors are constants during the deformation. Thus, the components of the Cauchy stress, the strain rate, and the spin need to be rotated as follows:

(16) $${\hat{\sigma }}_{0}=Q_{0}\sigma_{0}Q_{0}^{T}$$

(17) $$\hat{D}=Q_{\mathrm{Trial}}DQ_{\mathrm{Trial}}^{T}\;\;,\;\;\hat{W}=Q_{\mathrm{Trial}}WQ_{\mathrm{Trial}}^{T}$$

A return mapping algorithm is then used to determine the updated stress in the lattice frame. First, a trial stress is obtained with an elastic predictor:

(18) $${\hat{\sigma }}_{\mathrm{Trial}}={\hat{\sigma }}_{0}+\hat{C}:\hat{D}\Delta t,$$

where the fourth order tensor $\hat{C}$ denotes the constant elastic modulus with cubic symmetry in the lattice frame, characterized by only three independent material parameters $C_{11}$, $C_{12}$, and $C_{44}$. If the trial stress is inside the yield surface $f(\hat{\sigma })\leq 0$, then the material is elastic and the stress is the same as the trial stress. Otherwise, the deformation is plastic and the return mapping should be employed to calculate the stress. The yield surface is the key function of the plasticity model, and this time the Holmedal model [36] is chosen:

(19) $$f\left(\hat{\sigma }\right)=\varphi \left(\hat{\sigma }\right)-1={\left(\sum_{\alpha =1}^{N_{s}}{\left|\frac{\hat{\sigma }:{\hat{P}}^{\left(\alpha \right)}}{\tau_{c}^{\left(\alpha \right)}}\right|}^{n}\right)}^{\frac{1}{n}}-1=0,$$

where *n* is a material parameter. In Equation (19), the tensor ${\hat{P}}^{\left(\alpha \right)}$ is the symmetric part of the Schmid factor in the α slip system, and it is constant in the lattice frame. When plasticity, it is to solve the following system of nonlinear equations to obtain the values of $\hat{\sigma }$, $\dot{\lambda }$, and Γ:

(20a) $$\begin{array}{cc} \; & C^{-1}:\left({\hat{\sigma }}_{\mathrm{Trial}}-\hat{\sigma }\right)-\dot{\lambda }\frac{\partial \varphi }{\partial \hat{\sigma }}=0 \end{array}$$

(20b) $$\begin{array}{cc} \; & \varphi (\hat{\sigma },\Gamma )-1=0 \end{array}$$

(20c) $$\begin{array}{cc} \; & \left(\Gamma -\Gamma_{0}\right)-\Delta t\dot{\Gamma }\left(\hat{\sigma },\Gamma ,\dot{\lambda }\right)=0 \end{array}$$

In Equation (20b), the function φ depends on Γ because the critical resolved shear stress $\tau_{c}^{\left(\alpha \right)}$ in Equation (19) is a function of Γ. The Voce equation is chosen as the hardening law:

(21) $$\tau_{c}^{\left(\alpha \right)}=\tau^{\mathrm{ini}}+\Delta \tau^{\mathrm{sat}(\alpha )}\left(1-\exp\left(-\frac{\Gamma }{\Delta \gamma^{\mathrm{sat}(\alpha )}}\right)\right)$$

where $\tau^{\mathrm{ini}}$, $\Delta \tau^{\mathrm{sat}(\alpha )}$, and $\Delta \gamma^{\mathrm{sat}(\alpha )}$ are material parameters. In Equation (20b), the rate of the accumulated slip is calculated as follows:

(22) $$\dot{\Gamma }=\sum_{\alpha =1}^{N_{s}}\left|{\dot{\gamma }}^{\left(\alpha \right)}\right|,$$

where the rate of slip is calculated as follows:

(23) $${\dot{\gamma }}^{\left(\alpha \right)}=\frac{\dot{\lambda }}{\tau_{c}^{\left(\alpha \right)}}{\left|\frac{\hat{\sigma }:{\hat{P}}^{\left(\alpha \right)}}{\tau_{c}^{\left(\alpha \right)}}\right|}^{n-1}\mathrm{sign}\left(\hat{\sigma }:{\hat{P}}^{\left(\alpha \right)}\right).$$

The system of nonlinear equations is solved with Newton iterations including a line-search algorithm [18,37]. After the values of $\hat{\sigma }$, $\dot{\lambda }$, and Γ are obtained, the consistent algorithmic modulus ${\hat{C}}^{\mathrm{alg}}$ can be calculated in the lattice frame. The stress and the consistent algorithmic modulus should then be rotated back into the global frame by the updated orientation matrix Q:

(24) $$\sigma =Q^{T}\hat{\sigma }Q\;\;,\;\;C_{abcd}^{\mathrm{alg}}=Q_{ia}Q_{jb}Q_{mc}Q_{nd}{\hat{C}}_{ijmn}^{\mathrm{alg}}\;\;\left(\mathrm{in}\;\mathrm{the}\;\mathrm{index}\;\mathrm{notation}\right)$$

The orientation matrix is updated from the last value $Q_{0}$ as follows:

(25) $$Q={\left(I+\frac{\Delta t}{1+\frac{\Delta t^{2}}{4}{\hat{W}}^{c}:{\hat{W}}^{c}}\left({\hat{W}}^{c}+\frac{\Delta t}{2}{\hat{W}}^{c}{\hat{W}}^{c}\right)\right)}^{T}Q_{0}.$$

With an assumption of small elastic deformation, the co-rotated spin can be approximated as follows:

(26) $${\hat{W}}^{c}=\hat{W}-\sum_{\alpha =1}^{N_{s}}{\dot{\gamma }}^{\left(\alpha \right)}{\hat{\Omega }}^{\left(\alpha \right)}$$

where ${\hat{\Omega }}^{\left(\alpha \right)}$ is the skew-symmetric part of the Schmid tensor in the lattice frame. At last, the first Piola–Kirchhoff stress is obtained with Equation (33). The tangent stiffness K is calculated as done by Lucarini and Segurado [38], and the formulation for the components is

(27) $$\begin{array}{cc} K_{ijkl} & =J\left(C_{ipkm}^{\mathrm{alg}}F_{lm}^{-1}+\frac{1}{2}\left(\delta_{ik}F_{lm}^{-1}\sigma_{mp}-F_{li}^{-1}\sigma_{kp}+\delta_{pk}F_{lm}^{-1}\sigma_{im}-F_{lp}^{-1}\sigma_{ik}\right)\right)F_{jp}^{-1}\\ \; & -J\sigma_{ip}F_{lp}^{-1}F_{jk}^{-1}. \end{array}$$

#### 3.1.2. Calculation and Results

This stable but complicated constitutive model was originally coded in Fortran, serving as a user-defined material (UMAT) subroutine for commercial FEM codes. We coded the constitutive model in Python to perform the single-point calculation, taking advantage of the Python’s convenience in debugging and building. It is noteworthy that the Fortran subroutine can also be executed in the Python program, with the help of an interface already encapsulated in the package Numpy.

A uni-axial traction was simulated with the single-point calculator using this constitutive model. The plastic deformation preserves the volume, while the elastic deformation is not necessarily isochoric. For metals, the elastic deformation is small, so an incompressible uni-axial dilatation is often used in the strain control single-point calculation or one cubic element calculation. The prescribed values of the deformation gradient is often given as follows [26,27]:

(28) $$\bar{F}=\left[\begin{array}{ccc} \lambda & 0 & 0\\ 0 & \frac{1}{\sqrt{\lambda }} & 0\\ 0 & 0 & \frac{1}{\sqrt{\lambda }} \end{array}\right],$$

where λ>1, and its determinant J=1. However, in the case of anisotropic solids, the component $F_{22}$ is not necessarily identical to the component $F_{33}$, and the determinant is not necessarily 1. Therefore, a mixed control of the components of the stress and the deformation [25,39] should be used, as follows:

(29) $$\bar{F}=\left[\begin{array}{ccc} 1.4 & * & *\\ {}* & * & *\\ {}* & * & * \end{array}\right]\;\;,\;\;\bar{P}=\left[\begin{array}{ccc} {}* & 0 & 0\\ 0 & 0 & 0\\ 0 & 0 & 0 \end{array}\right].$$

**Figure 1:**
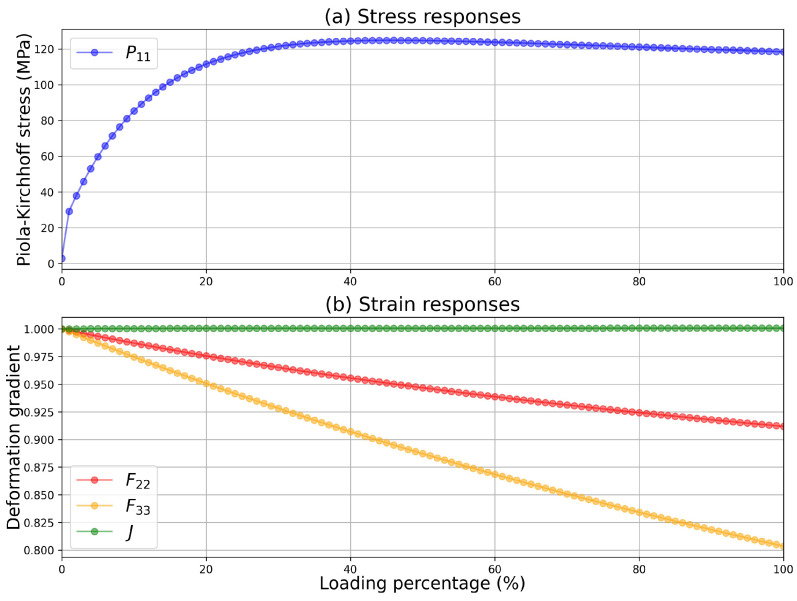
(**a**) The component $P_{11}$ of the Piola–Kirchhoff stress, (**b**) components $F_{22}$ and $F_{33}$ of the deformation gradient, and the determinant *J* as functions of the loading percentage under uni-axial traction. With the Euler angles ${[128.0}^{\circ },40.0^{\circ },37.0^{\circ }]$, components $F_{22}$ and $F_{33}$ have different values.

**Figure 2:**
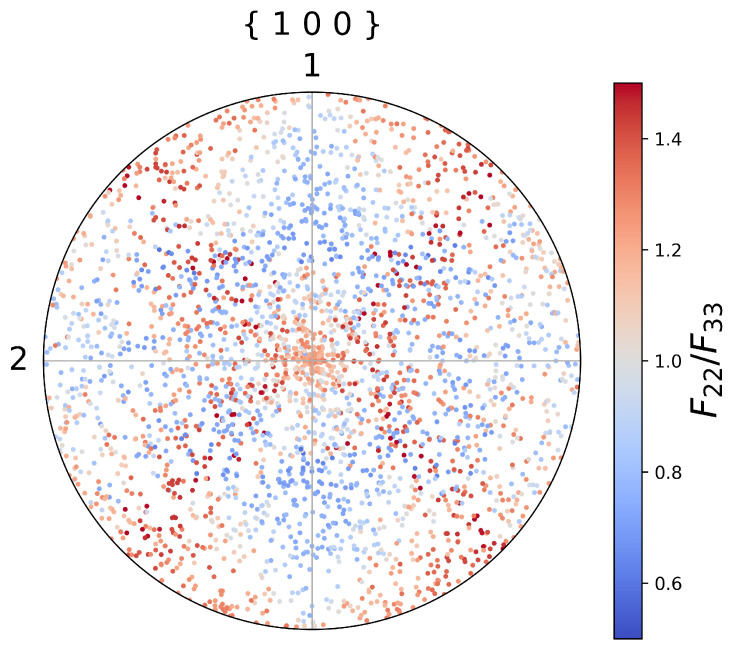
Distribution of the ratio of components $F_{22}$ to $F_{33}$ in the {100} pole figure under uni-axial traction when $F_{11}=1.4$. The positions of the points correspond to the randomly chosen Euler angles before deformations. The figure shows that the values of the ratio of $F_{22}$ to $F_{33}$ distribute differently when different initial Euler angles are chosen for the constitutive model.

With the material parameters in Table 1, the loading was simulated with the single-point calculator. After calculation, the Python program of the single-point calculator output the stress components and the deformation gradient, as well as all the internal variables. The components $F_{22}$, $F_{33}$, and the determinant *J* are depicted in Figure 1. From Figure 1, we can see that the determinant of the deformation gradient stays approximately at the value of 1, implying that the volume is nearly unchanged during deformations. The values of the components $F_{22}$ and $F_{33}$ decrease as the loading is gradually completed. A great difference between these two components $F_{22}$ and $F_{33}$ should not be ignored. This difference results from the anisotropic nature of the crystalline lattice, and this cannot be observed if the uni-axial dilatation as Equation (29) is applied in the strain control. This difference depends on the lattice orientation. Thus, 1000 randomly generated groups of Euler angles were investigated. The final values of the ratio of components $F_{22}$ to $F_{33}$ as functions of Euler angles are depicted in Figure 2. In Figure 2, we can observe the distributions of the values of the ratio of components $F_{22}$ to $F_{33}$, which is a reasonable result: as the solid described by this single crystal plasticity model is nearly isochoric, it is inferred that $F_{22}F_{33}\approx 1$, but $F_{22}\neq F_{33}$ under uni-axial traction for a given lattice orientation. This numerical phenomenon can be obtained easily with the proposed single-point calculator.

#### 3.1.3. Performance of the Calculator

The problem to solve for the performance of the single crystal plasticity model is highly nonlinear, as shown in Figure 1. The number of increment steps needs to be sufficient to avoid the issue of convergence. The minimum number of increments needed to converge for the numerical example presented in Section 3.1.2 was found to be 605 through the trial-and-error method. The performances of the single-point calculator was studied and compared with different total numbers of increments: 605, 1000, 2000, 5000, and 10,000.

**Figure 3:**
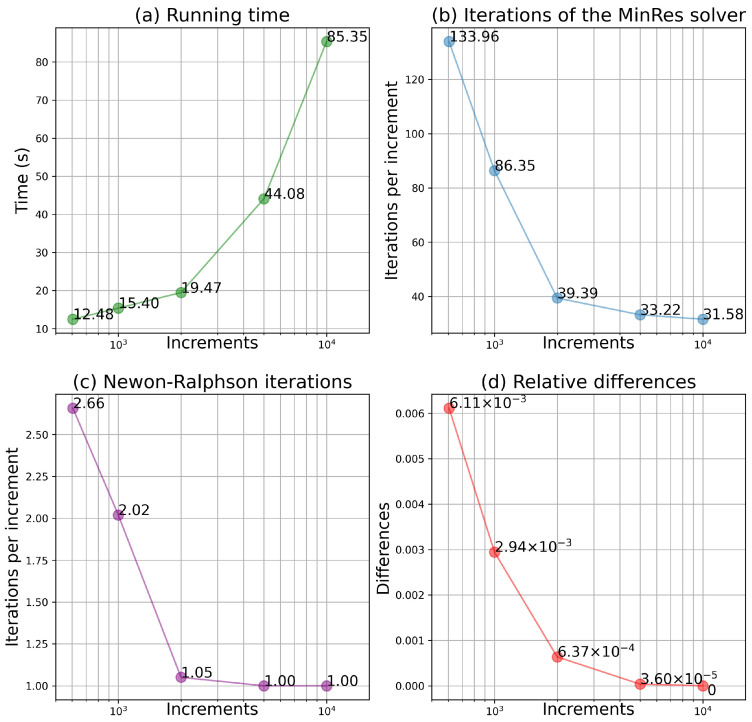
Performances of the single-point calculator as functions of the total number of increments for the simulation (presented in Section 3.1.2) with the single crystal plasticity model. The MinRes solver is chosen. The initial guess is the zero vector; the acceptable error is $1\times 10^{-8}$; the maximum number of iterations is 10,000.

Four dimensions of the performance are compared and shown in Figure 3. The total time needed to finish the simulation (as in Figure 3a) was recorded in a desktop computer with a 10-core CPU of 3.7 GHz. The total number of iterations for the MinRes solver and that of the Newtons were also recorded. The average numbers of iterations are calculated as the total number divided by the corresponding number of increments, shown in Figure 3b,c for the MinRes solver iterations and the Newton iterations, respectively. It is worth noting that the Newton iteration is used to solve nonlinear equations at one increment, while iterations for the Krylov solvers are needed to solve the system of linear equations, as shown in Equation (11), at each step in the Newton iteration. The last dimension is the relative difference, shown in Figure 3d. To calculate the differences, the deformation gradient and the first Piola–Kirchhoff stress calculated in 10,000 increments are chosen as the reference values, denoted as $F_{\mathrm{REF}}$ and $P_{\mathrm{REF}}$, respectively. The relative differences for each number of increments are calculated as follows:

(30) $$e=\frac{||F-F_{\mathrm{REF}}\left|\right|}{2||F_{\mathrm{REF}}\left|\right|}+\frac{||P-P_{\mathrm{REF}}\left|\right|}{2||P_{\mathrm{REF}}\left|\right|}$$

where ||·|| denotes the norm. The linearization the nonlinear expression of the stress (as in Equation (9)) implies a small increment of the deformation gradient. With a larger number of increments, the increment of the deformation gradient is smaller, so the results are more reliable, as shown in Figure 3d. In addition, smaller increments lead to a lower average number of iterations at one increment, as shown in Figure 3b,c. However, a higher computation cost is needed to achieve a better result, as shown in Figure 3d. In this case, the total number of increments of 2000 is recommended, as it can achieve an accuracy of about 99.95% of that with 10,000 increments, in less than one quarter of the time. In practical uses of the single-point calculator, an automatic stepping algorithm can be used to determine the magnitude of the increments at each step, to improve the efficiency of the calculator.

The choice of the Krylov solvers is important for the proposed single-point calculator. In Equation (11), the corresponding stiffness matrix of the system of linear equations, N:K:N, is possibly singular. For example, in the case of the mixed control as in Equation (29), the matrix representation of the stiffness is

$$\left[\begin{array}{ccccccccc} 0 & 0 & 0 & 0 & 0 & 0 & 0 & 0 & 0\\ 0 & K_{1212} & K_{1213} & K_{1221} & K_{1222} & K_{1223} & K_{1231} & K_{1232} & K_{1233}\\ 0 & K_{1312} & K_{1313} & K_{1321} & K_{1322} & K_{1323} & K_{1331} & K_{1232} & K_{1333}\\ 0 & K_{2112} & K_{2113} & K_{2121} & K_{2122} & K_{2123} & K_{2131} & K_{2132} & K_{2133}\\ 0 & K_{2212} & K_{2213} & K_{2221} & K_{2222} & K_{2223} & K_{2231} & K_{2232} & K_{2233}\\ 0 & K_{2312} & K_{2313} & K_{2321} & K_{2322} & K_{2323} & K_{2331} & K_{2332} & K_{2333}\\ 0 & K_{3112} & K_{3113} & K_{3121} & K_{3122} & K_{3123} & K_{3131} & K_{3132} & K_{3133}\\ 0 & K_{3212} & K_{3213} & K_{3221} & K_{3222} & K_{3223} & K_{3231} & K_{3232} & K_{3233}\\ 0 & K_{3312} & K_{3313} & K_{3321} & K_{3322} & K_{3323} & K_{3331} & K_{3332} & K_{3333} \end{array}\right],$$

with a vector representation of the increment of the deformation gradient as

$${\left[\delta {\tilde{F}}_{11},\delta {\tilde{F}}_{12},\delta {\tilde{F}}_{13},\delta {\tilde{F}}_{21},\delta {\tilde{F}}_{22},\delta {\tilde{F}}_{23},\delta {\tilde{F}}_{31},\delta {\tilde{F}}_{32},\delta {\tilde{F}}_{33}\right]}^{T}.$$

The bottom right non-zero components can be reformed into a new 8×8 matrix $K_{\mathrm{minor}}$. If the matrix $K_{\mathrm{minor}}$ is non-singular, Equation (11) can be solved with its inverse, which is the elimination method. There are two flaws. First, the size of the non-zero minor matrix $K_{\mathrm{minor}}$ depends on the stress control (*N* controlled components of stress lead to an N×N matrix). A problem-oriented treatment of the stiffness matrix without the generality of the solver is needed. Second, the minor matrix is not guaranteed to be non-singular. For example, at the beginning of the increment, the 8×8 matrix is singular as

$$\left[\begin{array}{cccccccc} 28340 & 0 & 28340 & 0 & 0 & 0 & 0 & 0\\ 0 & 28340 & 0 & 0 & 0 & 28340 & 0 & 0\\ 28340 & 0 & 28340 & 0 & 0 & 0 & 0 & 0\\ 0 & 0 & 0 & 106750 & 0 & 0 & 0 & 60410\\ 0 & 0 & 0 & 0 & 28340 & 0 & 28340 & 0\\ 0 & 28340 & 0 & 0 & 0 & 28340 & 0 & 0\\ 0 & 0 & 0 & 0 & 28340 & 0 & 28340 & 0\\ 0 & 0 & 0 & 60410 & 0 & 0 & 0 & 106750 \end{array}\right].$$

**Figure 4:**
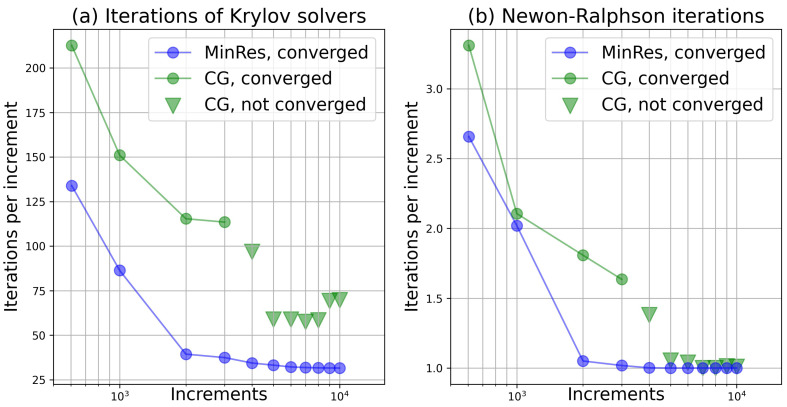
Performances of the MinRes and CG solvers in the single-point calculator as functions of the total number of increments for the simulation (presented in Section 3.1.2) with the single crystal plasticity model. The calculation is not converged with the CG solver when the number of increments is superior to 4000.

Despite the singularity, the system of Equations (Equation (11)) can be solved by Krylov solvers, such as CG or MinRes. The performances of CG and MinRes solvers are compared, as shown in Figure 4, from which we can see the advantage of the MinRes solver over the CG solver. In addition, an unexpected behavior of the CG solver can be observed that it does not converge when the total number of increments is superior to 4000. For the CG solver, N:K:N needs to transform to a tridiagonal matrix T by the Lanczos process. Then the Cholesky decomposition to T is applied. However, it is not always successful when N:K:N is singular. In this case, the CG solver is unstable. Different from CG, the MinRes solver finds approximated solutions to Equation (11) by minimizing the residual:

$$\left||r|\right|=||N:\left(\bar{P}-P\right)-N:K:N:\delta \tilde{F}\left|\right|.$$

A QR-decomposition of T through Givens rotations is performed, so that the residual decreases monotonically without divergence and stagnation, when N:K:N is singular [40]. With numerical experiments, it is found that the MinRes solver can guarantee the convergence of the Newton iterations, as long as the number of increment steps is sufficient. Thus, the MinRes solver is chosen as the solver of the system of linear Equations (Equation (11)) in the single-point calculator, due to its

- Unified form of Equation (11) for all kinds of the stress control;
- Ability to deal with the possible singular stiffness tangent;
- Robustness, comparing to the CG solver.

### 3.2. Application in Tests of an Artificial Neural Network Elasticity Model

As the second example, constitutive models developed with machine learning techniques for elasticity are tested with the single-point calculator. A black-box Artificial Neural Network (ANN) model [4] and a white-box Tensorial Sparse Symbolic Regressed (TSSR) model [13] are compared. A “black-box model” refers to the model relating the system inputs to the outputs with implicit calculations, whose arithmetic mechanism cannot be seen explicitly. On the contrary, a “white-box model” has an explicit arithmetic mechanism. These models are designed to model the macroscopic behavior of the hierarchical materials, fitting data generated from microscopic calculations.

#### 3.2.1. Data-Driven Constitutive Models

**Figure 5:**
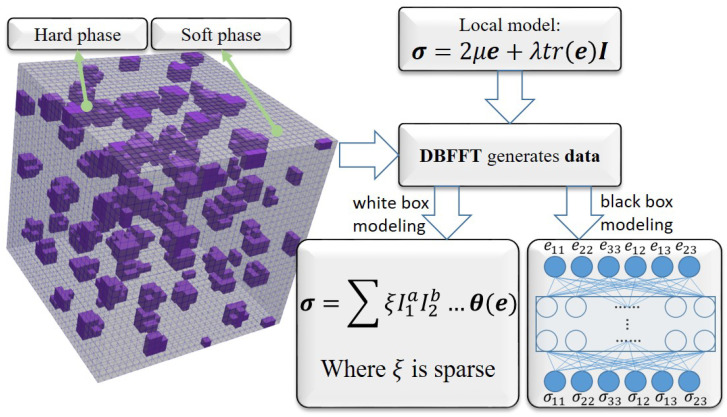
Representative volume element structure of the studied particle reinforced composite. The structure is represented in 31×31×31 voxels, 1333 of which are occupied by the reinforced phase and are distributed randomly and uniformly. Two phases of the structure are characterized by the local model. The macroscopic stress–strain data are calculated with the displacement-based-FFT (DBFFT) method. An artificial neural network (ANN) and a tensorial sparse symbolic regression (TSSR) model are trained with the data.

First, the calculation for generating stress–strain data is introduced. A particle reinforced composite (PRC) is considered, whose structure can be seen in Figure 5. The matrix phase (95.53% in volume) and the hard particle phase (4.47% in volume) can be characterized by the Eulerian linear relation between the Cauchy stress and the Almansi–Euler strain locally at each voxel:

(31) σ=2μe+λ(I:e)I,

where μ and λ are material parameters given in Table 2. The particles are distributed randomly and uniformly in the matrix. Fifty-seven different paths of deformation of the PRC were calculated with the displacement-based-FFT (DBFFT) algorithm [27], so more than 10,000 pairs of stress–strain data were generated for establishing macroscopic data-driven models.

The ANN model is used to establish an implicit mechanism to predict the components of the stress tensor with those of the strain tensor. The Cauchy stress and the left Cauchy–Green strain were chosen as output and input, respectively. As both the chosen stress and strain are symmetric, there are only six input nodes and six output nodes. Trained with the normalized data from DBFFT calculations, a multi-layer, fully connected ANN constitutive model with three hidden layers was established. The activation function is the hyperbolic tangent function. The number of nodes in each hidden layer was tuned as 30, 30, and 24 from input to output. This established an implicit relation model between the macroscopic Cauchy stress and the macroscopic left Cauchy–Green strain, as follows:

(32) $$\sigma =F_{\mathrm{ANN}}\left(B\right).$$

The Cauchy stress σ can be related to the first Piola–Kirchhoff stress as

(33) $$P=J\sigma F^{-T},$$

where *J* is the determinant of the deformation gradient F, J=det(F). The left Cauchy–Green deformation tensor B is calculated from the deformation gradient as

(34) $$B=FF^{T}.$$

For comparison, a TSSR model was also obtained with the same data. Even though TSSR is a data-driven method, it established an explicit expression of the relation between the stress and strain tensors:

(35) $$\begin{array}{cc} \sigma & =0.379J^{-2/3}I+0.0274J^{-3}I_{2}I-0.169J^{-8/3}I_{2}I-1.75J^{-2}I_{2}I\\ \; & +0.119J^{-7/3}I_{1}B+0.945J^{-2}I_{1}B+0.000186J^{-2}I_{2}^{2}B-0.107J^{-7/3}BB\\ \; & -0.957J^{-2}BB-7.02\times 10^{-7}J^{1/3}I_{2}^{2}BB+3.17I, \end{array}$$

where $I_{1}$ and $I_{2}$ are the first and second invariants of B, respectively. These two models were coded in Python and involved with the single-point calculator. For the ANN model, the offline training process was performed with the help of a Python machine learning package Scikit-learn [41]. The well established ANN model can be saved in a file after training. The constitutive model function in the single-point calculator then loads the ANN file to incorporate the ANN model, to predict the stress responses with given deformation gradients. As both the machine learning package and the calculator are Python-based, there were no additional efforts made to link the offline training and the calculating.

#### 3.2.2. Calculation and Results

In the case of stress or mixed control, the consistent tangent K is needed, as shown in Equation (11). For the TSSR model, the expression is already explicit, so the analytical expression of the tangent is easy to obtain. For multi-layer perceptrons, a kind of ANN with relatively simple structures, a consistent tangent can be constructed with the learned parameters, the layer structure information, and the derivatives of the activation functions [42,43]. However, in the general scope, it is hard to calculate the exact consistent tangent of an implicit and complicated network. A surrogate tangent can be learned with another ANN [44,45] from the data of the deformation gradient and the tangent calculated with DBFFT. One difficulty of this approach is that the tangent data is not always available. In addition, learning another ANN for the tangent is possibly very time-consuming. In our simple single-point calculator, a derivative-free approach is available with the central difference (CD) approximation of the derivatives [33,34]. The component $K_{ijmn}$ of the consistent tangent can be approximated as

(36) $$K_{ijmn}=\frac{P_{ij}\left(\left[F_{11},F_{12},...,F_{mn}+e,...,F_{33}\right]\right)-P_{ij}\left(\left[F_{11},F_{12},...,F_{mn}-e,...,F_{33}\right]\right)}{2e},$$

where *e* is a small scalar that is empirically taken as e=0.01/N (where N is the number of loading segment). With the help of the iterative Krylov solver, the single-point calculator can converge in the problem of stress and mixed control in using the CD approximated consistent tangent.

A mixed control was simulated successfully with the single-point calculator using these two data-driven constitutive models and the CD approximated consistent tangents. Components of the prescribed deformation gradient and the stress are given as follows:

(37) $$\bar{F}=\left[\begin{array}{ccc} 1.6 & 0 & 0\\ 0 & * & 0\\ 0 & 0 & * \end{array}\right]\;\;,\;\;\bar{P}=\left[\begin{array}{ccc} {}* & * & *\\ {}* & 0 & *\\ {}* & * & 0 \end{array}\right]$$

**Figure 6:**
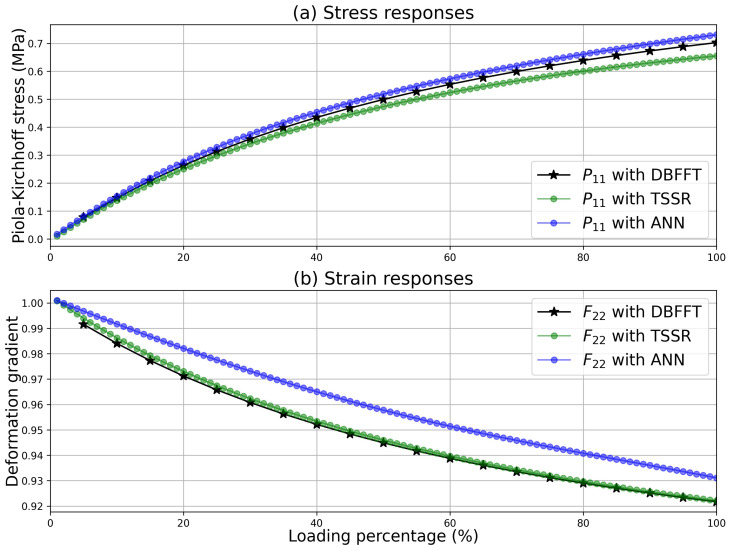
Stress and deformation gradient predictions with the trained ANN model, TSSR model, and the DBFFT full-field calculation. It shows (**a**) the component $P_{11}$ of the Piola–Kirchhoff stress and (**b**) the component $F_{22}$ of the deformation gradient as functions of the loading percentage.

The calculated components $P_{11}$ and $F_{22}$ are depicted in Figure 6. To evaluate the accuracy of the two surrogate models, the results calculated with DBFFT are also shown in Figure 6 as the reference values. Figure 6 shows that the trained ANN model predicts the increments of the stress components similarly to the TSSR model. Differences still exist between the stress results calculated with these two models, especially when the deformation is large. As for the deformation gradient, the differences from the reference are more significant for the ANN model than the TSSR model, from which we can argue that the established TSSR has a greater generalization ability than the ANN model. Nevertheless, calculations with these two models were easily made with the single-point calculator, facilitating fast tests, comparisons, and evaluations of these data-driven models.

## 4. Conclusions

In this work, we formulated a framework of single-point calculation for stress and mixed control to build, validate, and calculate with constitutive models. A projection operator was defined to mask the components of the deformation gradient and the stress, unifying the expressions of the equations for stress and mixed control boundary conditions. The Krylov solvers were chosen to solve the problem within the Newton iteration, as in the Galerkin–FFT methods. Python is easy to code and debug, and it is the most preferred language for well-known machine learning (e.g., Scikit-learn) or deep learning (e.g., TensorFlow or PyTorch) frameworks. Thus, the proposed single-point calculator and its demonstration constitutive models were coded in Python. Two examples of applications of the single-point calculator were then considered: a constitutive model with sophisticated mathematical structures for anisotropic single crystal plasticity and an artificial neural network constitutive model for elasticity. For implicit models whose analytical consistent tangents are hard to calculate, an alternative derivative-free method is available in the single-point calculator, with the central difference approximation method. It is illustrated that the proposed framework is suitable for developing and calculating with novel constitutive models, with structures either of a complicated explicit mathematical formula or of implicit multi-layer neural networks, for solid materials under large deformations, thus improving understanding and promoting the development of advanced materials. 

## Figures and Tables

**Table 1:** Material parameters used in the uni-axial traction simulation with the single crystal plasticity model.

Cubic Elasticity Modulus			Hardening Parameters			Euler Angles			
$C_{11}$ (GPa)	$C_{12}$ (GPa)	$C_{1}$ (GPa)	$\tau^{\mathrm{ini}}$ (MPa)	$\Delta \tau^{\mathrm{sat}(\alpha )}$ (MPa)	$\Delta \gamma^{\mathrm{sat}(\alpha )}$	$\theta_{1}$ (${}^{\circ }$)	$\theta_{2}$ (${}^{\circ }$)	$\theta_{3}$ (${}^{\circ }$)	n
106.75	60.41	28.34	10.0	63.0	0.1	128.0	40.0	37.0	100

**Table 2:** Coefficients of the constitutive model for the matrix and particles of the simulated particle reinforced composite.

Soft Matrix Phase		Hard Particle Phase	
λ (MPa)	μ (MPa)	λ (MPa)	μ (MPa)
1.5	1.0	7.5	5.0

## Data Availability

Not applicable.

## References

[1] Holzapfel G.A., Ogden R.W. (2010). Constitutive modelling of arteries. Proc. R. Soc. A Math. Phys. Eng. Sci..

[2] Cisse C., Zaki W., Zineb T.B. (2016). A review of constitutive models and modeling techniques for shape memory alloys. Int. J. Plast..

[3] Garcia-Gonzalez D., Hossain M. (2021). A microstructural-based approach to model magneto-viscoelastic materials at finite strains. Int. J. Solids Struct..

[4] Liu X., Tian S., Tao F., Yu W. (2021). A review of artificial neural networks in the constitutive modeling of composite materials. Compos. Part B Eng..

[5] Roters F., Eisenlohr P., Hantcherli L., Tjahjanto D., Bieler T., Raabe D. (2010). Overview of constitutive laws, kinematics, homogenization and multiscale methods in crystal plasticity finite-element modeling: Theory, experiments, applications. Acta Mater..

[6] Segurado J., Lebensohn R.A., LLorca J., Hussein M.I. (2018). Chapter One—Computational Homogenization of Polycrystals. Advances in Crystals and Elastic Metamaterials, Part 1.

[7] de Carvalho R., Valente R.A.F., Andrade-Campos A. (2010). On the Objective Function Evaluation in Parameter Identification of Material Constitutive Models - Single-point or FE Analysis. Int. J. Mater. Form..

[8] Kirchdoerfer T., Ortiz M. (2016). Data-driven computational mechanics. Comput. Methods Appl. Mech. Eng..

[9] Hartmaier A. (2020). Data-Oriented Constitutive Modeling of Plasticity in Metals. Materials.

[10] Kalayci C.B., Karagoz S., Karakas Ö. (2020). Soft computing methods for fatigue life estimation: A review of the current state and future trends. Fatigue Fract. Eng. Mater. Struct..

[11] Zhang P., Yin Z.Y., Jin Y.F. (2021). State-of-the-Art Review of Machine Learning Applications in Constitutive Modeling of Soils. Arch. Comput. Methods Eng..

[12] Logarzo H.J., Capuano G., Rimoli J.J. (2021). Smart constitutive laws: Inelastic homogenization through machine learning. Comput. Methods Appl. Mech. Eng..

[13] Wang M., Chen C., Liu W. (2022). Establish algebraic data-driven constitutive models for elastic solids with a tensorial sparse symbolic regression method and a hybrid feature selection technique. J. Mech. Phys. Solids.

[14] Huang Y. (1991). A User-Material Subroutine Incorporating Single Crystal Plasticity in the ABAQUS Finite Element Program.

[15] Harewood F., McHugh P. (2007). Comparison of the implicit and explicit finite element methods using crystal plasticity. Comput. Mater. Sci..

[16] Yasmeen T., Shao Z., Zhao L., Gao P., Lin J., Jiang J. (2019). Constitutive modeling for the simulation of the superplastic forming of TA15 titanium alloy. Int. J. Mech. Sci..

[17] Feng Z., Zecevic M., Knezevic M., Lebensohn R.A. (2022). Predicting extreme anisotropy and shape variations in impact testing of tantalum single crystals. Int. J. Solids Struct..

[18] Mánik T., Asadkandi H., Holmedal B. (2022). A robust algorithm for rate-independent crystal plasticity. Comput. Methods Appl. Mech. Eng..

[19] Rossiter J., Brahme A., Simha M., Inal K., Mishra R. (2010). A new crystal plasticity scheme for explicit time integration codes to simulate deformation in 3D microstructures: Effects of strain path, strain rate and thermal softening on localized deformation in the aluminum alloy 5754 during simple shear. Int. J. Plast..

[20] Zhang K., Hopperstad O., Holmedal B., Dumoulin S. (2014). A robust and efficient substepping scheme for the explicit numerical integration of a rate-dependent crystal plasticity model. Int. J. Numer. Methods Eng..

[21] Kohar C.P., Martin É., Connolly D.S., Patil S., Krutz N., Wei D., Inal K. (2019). A new and efficient thermo-elasto-viscoplastic numerical implementation for implicit finite element simulations of powder metals: An application to hot isostatic pressing. Int. J. Mech. Sci..

[22] Uchic M.D., Dimiduk D.M., Florando J.N., Nix W.D. (2004). Sample Dimensions Influence Strength and Crystal Plasticity. Science.

[23] Vondřejc J., Zeman J., Marek I. (2014). An FFT-based Galerkin method for homogenization of periodic media. Comput. Math. Appl..

[24] de Geus T., Vondřejc J., Zeman J., Peerlings R., Geers M. (2017). Finite strain FFT-based non-linear solvers made simple. Comput. Methods Appl. Mech. Eng..

[25] Wang M., Zhang K., Chen C. (2022). A mixed FFT-Galerkin approach for incompressible or slightly compressible hyperelastic solids under finite deformation. Comput. Methods Appl. Mech. Eng..

[26] Lucarini S., Segurado J. (2019). An algorithm for stress and mixed control in Galerkin-based FFT homogenization. Int. J. Numer. Methods Eng..

[27] Lucarini S., Segurado J. (2019). DBFFT: A displacement based FFT approach for non-linear homogenization of the mechanical behavior. Int. J. Eng. Sci..

[28] Lucarini S., Cobian L., Voitus A., Segurado J. (2022). Adaptation and validation of FFT methods for homogenization of lattice based materials. Comput. Methods Appl. Mech. Eng..

[29] Harris C.R., Millman K.J., van der Walt S.J., Gommers R., Virtanen P., Cournapeau D., Wieser E., Taylor J., Berg S., Smith N.J. (2020). Array programming with NumPy. Nature.

[30] Mozaffar M., Bostanabad R., Chen W., Ehmann K., Cao J., Bessa M.A. (2019). Deep learning predicts path-dependent plasticity. Proc. Natl. Acad. Sci. USA.

[31] Abadi M., Barham P., Chen J., Chen Z., Davis A., Dean J., Devin M., Ghemawat S., Irving G., Isard M. (2016). TensorFlow: A System for Large-Scale Machine Learning. Proceedings of the 12th USENIX Conference on Operating Systems Design and Implementation.

[32] Paszke A., Gross S., Massa F., Lerer A., Bradbury J., Chanan G., Killeen T., Lin Z., Gimelshein N., Antiga L. (2019). PyTorch: An Imperative Style, High-Performance Deep Learning Library. Proceedings of the 33rd International Conference on Neural Information Processing Systems.

[33] Berahas A.S., Byrd R.H., Nocedal J. (2019). Derivative-Free Optimization of Noisy Functions via Quasi–Newton Methods. SIAM J. Optim..

[34] Larson J., Menickelly M., Wild S.M. (2019). Derivative-free optimization methods. Acta Numer..

[35] Hughes T.J.R., Winget J. (1980). Finite rotation effects in numerical integration of rate constitutive equations arising in large-deformation analysis. Int. J. Numer. Methods Eng..

[36] Holmedal B. (2020). Regularized Yield Surfaces for Crystal Plasticity of Metals. Crystals.

[37] Scherzinger W. (2017). A return mapping algorithm for isotropic and anisotropic plasticity models using a line search method. Comput. Methods Appl. Mech. Eng..

[38] Lucarini S., Segurado J. (2018). On the accuracy of spectral solvers for micromechanics based fatigue modeling. Comput. Mech..

[39] Ma R., Truster T.J. (2019). FFT-based homogenization of hypoelastic plasticity at finite strains. Comput. Methods Appl. Mech. Eng..

[40] Tinzefte A., Menach Y.L., Piriou F. (2009). Iterative Solvers for Singular Symmetric Linear Systems in Low Frequency Electromagnetics. IEEE Trans. Magn..

[41] Pedregosa F., Varoquaux G., Gramfort A., Michel V., Thirion B., Grisel O., Blondel M., Prettenhofer P., Weiss R., Dubourg V. (2011). Scikit-learn: Machine Learning in Python. J. Mach. Learn. Res..

[42] Ratku A., Neumann D. (2022). Derivatives of feed-forward neural networks and their application in real-time market risk management. OR Spectr..

[43] Rodini S. (2022). Analytical derivatives of neural networks. Comput. Phys. Commun..

[44] Im S., Kim H., Kim W., Cho M. (2020). Neural network constitutive model for crystal structures. Comput. Mech..

[45] Gu Q., Pan J., Liu Y., Fu M., Zhang J. (2022). An Effective Tangent Stiffness of Train–Track–Bridge Systems Based on Artificial Neural Network. Appl. Sci..

